# Efficacy and Effectiveness of Carnitine Supplementation for Cancer-Related Fatigue: A Systematic Literature Review and Meta-Analysis

**DOI:** 10.3390/nu9111224

**Published:** 2017-11-07

**Authors:** Wolfgang Marx, Laisa Teleni, Rachelle S. Opie, Jaimon Kelly, Skye Marshall, Catherine Itsiopoulos, Elizabeth Isenring

**Affiliations:** 1 School of Allied Health, College of Science, Health and Engineering, La Trobe University, Melbourne, VIC 3086, Australia; R.Opie@latrobe.edu.au (R.S.O.); C.Itsiopoulos@latrobe.edu.au (C.I.); 2 Faculty of Health Sciences & Medicine, Bond University, Gold Coast, QLD 4226, Australia; laisa.teleni@student.bond.edu.au (L.T.); jkelly@bond.edu.au (J.K.); smarshal@bond.edu.au (S.M.); lisenrin@bond.edu.au (E.I.)

**Keywords:** carnitine, fatigue, cancer, dietary supplement, systematic review

## Abstract

Background: Carnitine deficiency has been implicated as a potential pathway for cancer-related fatigue that could be treated with carnitine supplementation. The aim of this systematic literature review and meta-analysis was to evaluate the literature regarding the use of supplemental carnitine as a treatment for cancer-related fatigue. Methods: Using the PRISMA guidelines, an electronic search of the Cochrane Library, MEDLINE, Embase, CINAHL and reference lists was conducted. Data were extracted and independently assessed for quality using the Academy of Nutrition and Dietetics evidence analysis by two reviewers. In studies with positive quality ratings, a meta-analysis was performed using the random-effects model on Carnitine and cancer-related fatigue. Results: Twelve studies were included for review with eight reporting improvement in measures of fatigue, while four reported no benefit. However, many studies were non-randomized, open-label and/or used inappropriate dose or comparators. Meta-analysis was performed in three studies with sufficient data. Carnitine did not significantly reduce cancer-related fatigue with a standardized mean difference (SMD) of 0.06 points ((95% CI −0.09, 0.21); *p* = 0.45). Conclusion: Results from studies with lower risk of bias do not support the use of carnitine supplementation for cancer-related fatigue.

## 1. Introduction

Cancer-related fatigue (CRF) is one of the most common side-effects of cancer treatment, affecting up to 91% of patients and is now considered one of the most distressing symptoms of cancer [1,2]. CRF is associated with worsened quality of life, depression and anxiety, inability to perform activities of daily living and can have significant financial costs to both patients and their caregivers [3,4]. Furthermore, CRF can persist for months or years after the completion of cancer treatment and is associated with reduced recurrence-free and overall survival [5,6,7,8].

Despite its high prevalence, the aetiology of CRF remains unclear. Mechanisms implicated in the development of CRF include inflammation, anaemia and altered neuroendocrine pathways [9]. In addition, most observational studies report that decreased serum carnitine has been associated with fatigue [10,11,12,13]. Carnitine is pivotal to energy production; facilitating the uptake of fatty acids into the mitochondria for beta-oxidation. Chemotherapy regimens including cisplatin and ifosfomide disrupt carnitine metabolism by increasing the renal excretion of carnitine [14,15]. Furthermore, muscle wasting, as seen in cancer cachexia, can further exacerbate fatigue. Carnitine may have multiple properties that can prevent or reduce muscle wasting including modulation of protein synthesis and degradation, as well as anti-apoptotic, antioxidant and anti-inflammatory properties [16]. Therefore, it is plausible that the restoration of serum carnitine levels via supplemental carnitine could ameliorate CRF.

Several trials have investigated the effectiveness of supplemental carnitine for the management of CRF. The aim of this review was to systematically evaluate intervention trials regarding the use of supplemental carnitine as a treatment for cancer-related fatigue to inform the clinical management of CRF.

## 2. Methods

This study was prepared in accordance with the Preferred Reporting Items for Systematic Reviews and Meta-Analyses (PRISMA) guidelines [17].

### 2.1. Eligibility Criteria

Eligible studies included those evaluating CRF as a primary or secondary outcome in patients of any cancer diagnosis and age, supplemented with carnitine as either a stand-alone intervention or in combination with other agents. Studies were limited to those published in English. Randomized and pseudo-randomized controlled trials were preferred; however, if there were less than five randomized studies, non-randomized and single arm studies were included.

### 2.2. Data Collection and Extraction

The CINAHL, Cochrane Library (i.e., Cochrane CENTRAL and Cochrane Database of Systematic Reviews), Embase and MEDLINE databases were searched from database inception to December 2016. Two reviewers (Wolfgang Marx and Laisa Teleni) independently screened the titles and abstracts for relevance. Relevant articles were retrieved and two review authors (Wolfgang Marx and Laisa Teleni) independently screened the full text for eligibility. The reference lists of eligible articles were screened for relevant publications. Data extraction was performed in duplicate (Wolfgang Marx and Rachelle S Opie) and a third author (Liz Isenring) was designated as referee.

Extracted data included the study design, inclusion and exclusion criteria, patient demographics (e.g., age, gender), dosing schedule (including dose and frequency), sample size (including dropout rates and reasons), method used to assess fatigue, study outcomes (including self-reported measures of fatigue and adverse events), funding details and potential conflict of interest. We extracted the mean (change from baseline or end-of-study value) and appropriate variance data (standard deviation, standard error or 95% confidence intervals) to perform the meta-analysis.

### 2.3. Assessment of Study Quality

The quality of the included studies was assessed independently by two authors (Wolfgang Marx and Rachelle S Opie) using the Academy of Nutrition and Dietetics Quality Criteria Checklist which assesses studies for selection, allocation, reporting and attrition bias as well as the level of external validity. Using this checklist, each study was assigned a positive (low risk of bias), negative (high risk of bias) or neutral (moderate risk of bias) rating.

### 2.4. Data Synthesis

Studies that were rated as positive quality were pooled into Revman for meta-analysis using the DerSimonian and Laird random-effects model [18,19]. Based on the quality rating of the included studies, meta-analysis was deemed inappropriate in 9 of the 12 studies and therefore, findings are presented in narrative form. Treatment effect was calculated as the standardized mean difference (SMD) due to the variability in fatigue measurement scales. One study was standardized to a negative score by multiplying the mean and standard deviation by −1 to reflect a directional score consistent with the other studies [20,21]. A statistically significant (*p* < 0.05) result was considered evidence of an effect.

## 3. Results

### 3.1. Study Characteristics

**Figure 1:**
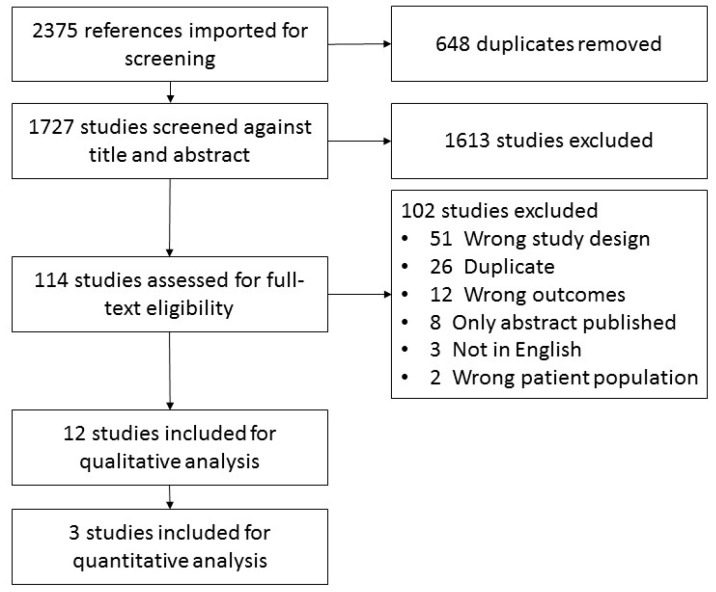
Systematic review flow diagram.

Of the 1727 articles screened, 12 met the eligibility criteria (Figure 1). Population groups included eight advanced cancer, seven mixed diagnoses, two breast cancer only and the remaining studies included pancreatic, gynaecological cancer or multiple myeloma patients only. Three studies included patients with carnitine deficiency only (as confirmed by blood test), five studies included patients with self-reported fatigue and six had no restriction on carnitine deficiency or fatigue in their eligibility criteria.

The included studies used a variety of study designs. Three studies were single-arm trials and eight studies used a comparator arm, four of which used a placebo control while five used either standard care or various active ingredients (see Table 1). Eight studies were open-label, two studies were double-blinded throughout the intervention [20,22] and two studies incorporated both a double-blinded and an open-label phase [23,24]. Intervention characteristics and key findings are summarized in Table 1.

There was a large range of sample sizes with eight studies having a total sample size below 100 participants. However, four studies had moderate-to-large sample sizes that ranged from 144 to 409 participants [20,24,25,26].

### 3.2. Carnitine Regimens

All studies used an oral dose of carnitine, delivered in a range of forms including liquid (*n* = 5) [22,23,24,27,28] capsule (*n* = 1) and jelly (*n* = 1) formulations [29]. Nine studies used a dose between 2 to 6 g per day and three studies used <2 g/day, taken once or up to three divided doses per day, with Iwase et al. [29] using the smallest dose of 50 mg [23,27]. Most studies used carnitine as a stand-alone intervention—however, three studies used carnitine as a co-intervention with antioxidant supplements (e.g., coenzyme Q10, alpha lipoic acid), nonsteroidal anti-inflammatories (e.g., celecoxib) and steroids (e.g., megestrol acetate and medroxyprogesterone acetate) [25,29,30]. The intervention period varied from one week up to 24 weeks.

### 3.3. Outcome Measures

All studies used a self-reported measure to assess fatigue with most studies using the following validated questionnaires: The Multidimensional Fatigue Symptom Inventory—Short Form, Functional Assessment of Chronic Illness Therapy-Fatigue Scale, or the Brief Fatigue Index (BFI). Three studies calculated sample sizes *a priori* based on fatigue with one study, Cruciani et al. [24], achieving the required sample size [23,24,29]. Five studies did not report on fatigue as their primary outcome but provided powered calculations based on other outcomes (e.g., lean body mass, peripheral neuropathy and inflammation) and four provided no sample size calculation.

Other secondary outcomes included quality of life, anthropometric measures (e.g., lean body mass, grip strength, DEXA), pathology (e.g., reactive oxygen species, glutathione peroxidase, superoxide dismutase, pro-inflammatory cytokines and C-reactive protein), physical function, depression and mood scales, measures of peripheral neuropathy and treatment response (e.g., complete remission, partial response, minimal response).

### 3.4. Compliance Measures

Five studies reported on compliance with Hershman et al. [20] (pill count) and Kraft et al. [22] (serum carnitine) providing sufficient detail on the method of measuring compliance [25,26,30].

### 3.5. Quality Rating

Three studies included in this review received a positive quality rating with two receiving a neutral rating and seven receiving a negative rating (Table 1). The primary reasons for the neutral or negative rating were a lack of blinding, lack of inclusion of a placebo/control group, lack of adjustment of potential confounders and randomization, failure to conduct an intention-to-treat analysis.

### 3.6. Intervention Results on Cancer-Related Fatigue

Four studies reported no improvement in measures of CRF in response to carnitine supplementation and eight studies reported significant improvement in CRF (Table 2). Four single arm studies reported significant improvements when compared to baseline [27,28,30,31]. Iwase et al. [29] reported a significant improvement in worst level of and mean change in fatigue compared to the control group but not average fatigue. Maccio et al. [25] reported that a combination therapy that included carnitine (as well as celecoxib, alpha lipoic acid, carboxycysteine and megestrol acetate) resulted in a significantly improved CRF compared to the control group. Similarly, Mantovani et al. [26] reported a significant improvement in fatigue when using a combination intervention but not when participants received carnitine and an antioxidant supplement only. Cruciani et al. [23] found no significant difference between the intervention and placebo group during a blinded phase; however, after a second, open-label phase whereby all participants received the carnitine supplement, the participants who were originally allocated to the blinded intervention group reported significantly improved levels of fatigue.

All studies with a low risk of bias reported no significant difference in measures of fatigue while most studies with a moderate to high risk of bias reported significant improvements in fatigue.

### 3.7. Adverse Events

Eight studies provided data on adverse events. Commonly reported adverse events included diarrhoea (*n* = 7 studies) [20,23,24,25,26,29,32] and haematological toxicities (*n* = 3 studies) [24,29,32] with both symptoms reported in approximately less than five patients in each study reporting ≥grade 3 symptoms. Of the studies that included a control group, no significant increase in adverse events in the intervention group was reported.

### 3.8. Meta-Analysis

**Figure 2:**
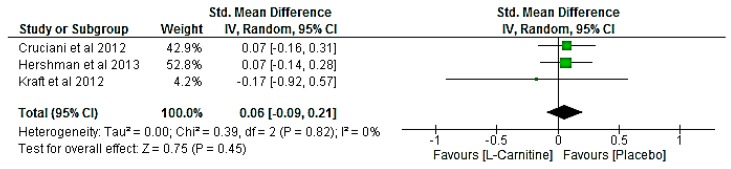
Forest plot of the effect of Carnitine dietary supplement on Cancer-related fatigue. CI = confidence interval; IV = inverse variance.

In three studies involving a total of 659 participants [20,22,24], carnitine did not significantly reduce CRF (SMD of 0.06 points (95% CI −0.09, 0.21); *p* = 0.45; Figure 2). There was no evidence of statistical heterogeneity (*I*^2^ = 0%). Clinical heterogeneity was evident from the three studies in regards to the dose (2–4 g of carnitine per day), patient demographics (40–100% females included) and carnitine status. However, there were not enough studies to conduct sensitivity analyses to isolate these potential sources of heterogeneity and test the robustness of findings.

## 4. Discussion

Our review identified 12 studies that investigated the use of orally administered carnitine for the treatment of CRF. Despite most (8/12) studies reporting a significant improvement in measures of CRF, most (9/12) studies contain significant limitations that require consideration. Many studies were open-label, single-arm trials which introduces significant performance, selection and detection bias, particularly for fatigue, a self-reported measure that is likely to be susceptible to a placebo response. Some studies either did not analyse or did not find statistical improvements in fatigue when compared to a control or other intervention groups and instead reported improvements in fatigue at the final time point when compared to baseline. The lack of significant improvements compared to a parallel control group further limits the confidence that these improvements are attributed to the intervention alone.

The two largest studies, both randomized controlled trials, reported no significant difference in CRF between intervention and control groups [20,24]. Hershman et al. [20] reported no significant difference in fatigue; however, CRF was a secondary outcome and did not exclusively recruit patients reporting carnitine deficiency or fatigue. The only included study that was sufficiently powered to detect significant differences in fatigue, Cruciani et al. [24], found no effect in any measure of fatigue despite promising results from their earlier studies [27,33]. A possible explanation for these differences is that earlier studies from the same authors recruited carnitine deficient patients only (defined as free carnitine <35 mM/L for males or <25 mM/L for females, or acyl/free carnitine ratio >0.4) which is in contrast to their largest study which recruited patients with moderate to severe fatigue, irrespective of carnitine status [24]. However, an included subgroup analysis of carnitine deficient patients reported no statistically significant differences in CRF despite mean CRF levels in the carnitine group being consistently lower at follow-up time points. The authors noted that the dose used in their study was lower than doses used in studies that have reported improvements in fatigue (1 g versus up to 6 g) [25]. However, there are also studies that have investigated higher doses that have reported no significant improvements [20,22,32].

While the lower doses of carnitine used in some included studies (e.g., 50 mg reported by Iwase et al. [29]) are unlikely to deliver a sufficient dose of absorbable carnitine to provide a therapeutic effect, pharmacokinetic research suggests that higher doses may also be suboptimal [34,35]. Carnitine supplementation has relatively poor oral bioavailability, with 5–16% of carnitine being absorbed after a single dose of 2 g and 6 g of carnitine in healthy participants [34]. Furthermore, plasma concentrations of carnitine are non-linear with one study reporting that doses of 0.5 g, 1 g and 2 g resulted in similar plasma concentrations of carnitine while the 2 g dose resulted in significantly increased plasma concentrations of a proatherogenic metabolite of carnitine, trimethylamine-*N*-oxide [36], indicating that absorption had been saturated and that doses exceeding 2 g may not provide further therapeutic effect [35]. It should be noted, however, that the cited pharmacokinetic studies have all been conducted in healthy participants and the dose of carnitine required to saturate absorption pathways may differ in carnitine deficient populations.

There are multiple risk factors for CRF including but not limited to, inflammation, anaemia and altered neuroendocrine pathways [9]. The lack of control for these potential mechanisms of fatigue in the included studies may have confounded the results. This is partially supported by the positive results of studies that investigated carnitine as part of a multi-ingredient intervention that targeted multiple pathways [25,26,30].

Follow-up periods and patient populations were also inconsistent across studies. Carnitine status fluctuates both during the course of cancer treatment and in response to different chemotherapy regimens [11,12]. For example, doxorubicin is reported to affect carnitine levels to a greater extent than other treatments and Heuberger et al. [37] reported carnitine levels to increase one week after chemotherapy while studies that have measured carnitine status with longer time points have reported a decrease [11,12]. Hence, future studies are required to investigate carnitine fluctuations over the course of chemotherapy treatment and intervention trials may benefit from recruiting homogenous cancer populations.

### Future Directions and Clinical Implications

Due to the dearth of clinical studies with low risk of bias, clinical recommendations for the use of carnitine in treating CRF are premature. Only 3 of the 12 studies were suitable for meta-analysis which reduces the conclusions which can be drawn from the analysis. The available evidence indicates carnitine supplementation is unlikely to provide a clinically meaningful benefit for CRF in the chemotherapy cancer-population.

Further research is required to elucidate the safety profile of carnitine in the cancer setting. While the included studies reported carnitine supplementation to be well tolerated, Hershman et al. [20] found symptoms of peripheral neuropathy increased in participants receiving carnitine supplementation, which should be considered before clinical use. Furthermore, carnitine supplementation may increase risk of cardiovascular disease via the increase in proatherogenic microbiota-derived metabolites trimethylamine, trimethylamine-*N*-oxide and γ-butyrobetaine [36] These metabolites have also been shown in animal models to increase concentrations of the carcinogenic compound *N*-nitrosodimethylamine [38]. Therefore, long term carnitine supplementation may increase risk of chronic diseases, particularly at higher doses which provides a greater amount of substrates for these proatherogenic metabolites.

Biases inherent in intervention study designs with high-risk of bias mean it is difficult to determine if the reported adverse events can be attributed solely to carnitine supplementation. Future intervention studies should utilize trial designs with the lowest risk of bias (e.g., randomized controlled trials), implement methods of measuring adherence to intervention (e.g., pill counts and/or serum carnitine), investigate carnitine as a stand-alone intervention and ensure study populations are controlled for aetiology of fatigue and chemotherapy regimens.

## 5. Conclusions

Of the 12 studies included in this systematic review, eight reported carnitine supplementation to significantly improve measures of cancer-related fatigue. However, due to the significant bias of many included studies, the null findings of the two largest studies and our meta-analysis and the potential increase in peripheral neuropathy, there is currently insufficient evidence to recommend its use in the cancer setting. Future studies should include rigorous study design methods to reduce bias and focus on population subsets with confirmed carnitine deficiency.

## Figures and Tables

**Table 1:** Study Design, Population and Quality of Included Studies.

Study & Design	Study Design and Quality	Population and Attrition	Sample Size and Attrition
Graziano et al. 2002 [31]	Single arm intervention study Study quality: Negative COI: none stated Funding: none reported	Advanced cancer. Undergoing first line, palliative chemotherapy. Mixed cancer diagnoses Mean age: 61 (range: 45–70) years Female: 40%	*N* = 50 Attrition: 0% Withdrawal reasons: N/A
Gramignano et al. 2006 [28]	Open-label, single-arm intervention study Study quality: Negative COI: none stated Funding: none reported	Advanced cancer. Mixed cancer diagnoses Mean age: 60 ± 9 years Female: 83%	*N* = 12 Attrition: 0% Withdrawal reasons: N/A
Cruciani et al. 2006 [27]	Open-label, Single-arm intervention study Study quality: Negative COI: none stated Funding: none reported	Carnitine deficient. Advanced cancer. Mixed cancer diagnoses Mean age: 60 ± 14 years Female: 37%	*N* = 27 (*n* = 3 to 6 per dosage group) Attrition: 22% (*n* = 3 per 7 dosage groups) Withdrawal reasons: hospitalization (*n* = 2), severe deterioration (*n* = 3) and protocol violation (*n* = 1)
Callander et al. 2014 [32]	Open-label, non-randomised controlled trial Study quality: Negative COI: none stated Funding: Partially funded by an unrestricted grant from Millennium	Relapsed and/or refractory multiple myeloma Mean age: 65 ± 12 years Female: 34%	*N* = 32 (*n* = 13 IG; *n* = 19 CG) Attrition: 16% (*n* = 13 IG; *n* = 14 CG) IG: withdrawal reasons: unclear. CG withdrawal reasons: LFT abnormality (*n* = 1); deterioration (*n* = 4)
Iwase et al. 2016 [29]	Open-label RCT Study quality: Negative COI: none stated Funding: Otsuka Pharmaceutical Factory Incorporated	Women with diagnosed breast cancer Median age: 49–52 (range: 22–70) years Female: 100%	*N* = 59 (*n* = 28 IG; *n* = 31 CG) Attrition: 3% (*n* = 59 IG; *n* = 29 CG) IG: withdrawal reasons: N/A CG withdrawal reasons: declined (*n* = 1); deterioration (*n* = 1)
Cruciani et al. 2009 [30]	Double-blind, placebo-controlled randomized trial; with control-group open-label cross-over Study quality: Negative COI: none stated Funding: none reported	Carnitine deficient. Advanced cancer. Mixed cancer diagnoses Mean age: 66–70 ± 13 years Female: 55%	*N* = 33 (*n* = 27 IG including *n* = 10 from CG cross-over; *n* = 12 CG) Attrition: 36% (*n* = 10 IG; *n* = 7 CG) IG withdrawal reasons: died (*n* = 2), deterioration (*n* = 3), diarrhoea (*n* = 1), missed follow-up (*n* = 1) CG withdrawal reasons: died (*n* = 1), deterioration (*n* = 2), fatigue (*n* = 2)
Kraft et al. 2012 [22]	Double-blind, placebo-controlled randomized trial Study quality: Positive COI: none stated Funding: unrestricted educational grants from Medinal GmbH, Greven, Germany, Fresenius Kabi Germany GmbH Bad Homburg, Germany and Nutricia GmbH, Erlangen, Germany	Stage IV Pancreatic cancer Mean age: 64 ± 2 years Female: 40%	*N* = 72 (*n* = 38 IG; *n* = 34 CG) Attrition: 64% (*n* = 14 IG; *n* = 12 CG) Withdrawal reasons (groups reported together, no significant difference: died (*n* = 2), deterioration (*n* = 3), diarrhoea (*n* = 1), missed follow-up (*n* = 1)
Cruciani et al. 2012 [24]	Double-blind placebo-controlled randomized trial with control-group cross-over Study quality: Positive COI: none stated Funding: Financial support contributed by Ricardo A. Cruciani (lead author)	Mixed cancer diagnoses Age not reported Female: 58%	*N* = 376 (*n* = 198 IG; *n* = 187 CG) Attrition: 44% (*n* = 104 IG; *n* = 105 CG) IG: Withdrawal reasons: died (*n* = 8), refused treatment (*n* = 37), deterioration (*n* = 7), became ineligible (*n* = 24), adverse events (*n* = 2), others (*n* = 7) CG withdrawal reasons: died (*n* = 8), refused treatment (*n* = 30), deterioration (*n* = 5), adverse events (*n* = 29), others (*n* = 9)
Hershman et al. 2013 [20]	Double-blind placebo-controlled randomized trial Study quality: Positive COI: none stated Funding: Financial support by Dawn L. Hershman	Women with diagnosed breast cancer undergoing taxane-based adjuvant chemotherapy Median age: 50–52 (range: 26–80) years Female: 100%	*N* = 409 (*n* = 208 IG; *n* = 201 CG) Attrition: 3% (*n* = 201 IG; *n* = 194 CG) Withdrawal reasons: not described
Mantovani et al. 2010 [26]	Open-label, five-arm randomized non-controlled trial Study quality: Neutral COI: none stated Funding: provided by lead author	Advanced cancer. Cancer-related anorexia/cachexia. Mixed cancer diagnoses Mean age: 63 ± 12 years Female: 47%	*N* = 332 (*n* = 88 IG-a (l-carnitine alone); *n* = 88 IG-b (l-carnitine + other therapies); *n* = 156 other groups) Attrition: 3% (*n* = 85 IG-a; *n* = 86 IG-b) IG-a withdrawal reasons: died (*n* = 3) IG-b withdrawal reasons: died (*n* = 2)
Macciò et al. 2012 [25]	Open-label, two-arm randomized non-controlled trial Study quality: Neutral COI: none stated Funding: none stated	Gynaecological cancer only. Advanced cancer. Cancer-related anorexia/cachexia Mean age: 61 ± 13 years Female: 100%	*N* = 144 (*n* = 72 IG-a (l-carnitine + other therapies); *n* = 72 IG-b (MA alone)) Attrition: 14% (*n* = 61 IG-a; *n* = 63 IG-b)IG-a withdrawal reasons: died (*n* = 8), poor compliance (*n* = 3) IG-b withdrawal reasons: died (*n* = 7), poor adherence (*n* = 2)
Madeddu et al. 2012 [30]	Open-label, two-arm randomized non-controlled trial Study quality: Negative COI: not reported Funding: none stated	Advanced cancer. Cancer-related anorexia/cachexia. Mixed cancer diagnoses Mean age: 65 ± 9 years Female: 30%	*N* = 60 (*n* = 31 IG-a (l-carnitine + celecoxib); *n* = 29 IG-b (l-carnitine + celecoxib + MA)) Attrition: 7% (*n* = 29 IG-a; *n* = 27 IG-b) IG-a withdrawal reasons: died (*n* = 2) IG-b withdrawal reasons: died (*n* = 2)

CG, control group; COI, conflict of interest; IG, intervention group; MA, N/A, not applicable.

**Table 2:** Intervention and Results of Included Studies.

Study & Design	Intervention	Results
Graziano et al. 2002 [31]	Intervention: Levocarnitine supplement, not further described Comparator: None Dose: 2 g × 2 per day (4 g total per day) Duration: 1-week	**Fatigue:** At 3-weeks post-baseline: *FACT-Fatigue* (scored 0–65; lower scores indicate more severe symptoms) μ 36.5 ± 5.1; mean change from baseline 1.6 (*P* > 0.05 since baseline)
Gramignano et al. 2006 [28]	Intervention: Levocarnitine solution Comparator: none Dose: 2 g × 3 per day (6 g total per day) Duration: 4-weeks	**Fatigue:** At 4-weeks post-baseline: *MFSI-SF* (scored 0–150; higher scores indicate more severe symptoms) μ 12.1 ± 12.6; mean change from baseline −13.3 (***P* < 0.001 since baseline**) **Quality of Life:** At 4-weeks post-baseline: *QoL-OS* (scored 0–73; higher scores indicate more severe symptoms) μ 36.8 ± 15.7; mean change from baseline −17.5 (***P* < 0.05 since baseline**) *EQ-5D Visual Analogue Scale* (scored 0–100; lower scores indicate more severe symptoms) μ 73.3 ± 12.4; mean change from baseline 22.7 (*P* < 0.001 since baseline) **Anthropometry:** At 4-weeks post-baseline: *Lean body mass* via BIA μ 40.4 ± 8.6kg; mean change from baseline 2.4 kg (***P* < 0.05 since baseline**) **Appetite:** At 4-weeks post-baseline: *Numerical scale* (scored 0–10; lower scores indicate more severe symptoms) μ 6.8 ± 1.9; mean change from baseline 2 (***P* = 0.001 since baseline**) **Muscle strength:** At 4-weeks post-baseline: *Grip strength* via dynamometer data not reported (*P* > 0.05 since baseline) **Pathology:** At 4-weeks post-baseline: *ROS* μ 415.2 ± 126.0 FORT units; mean change from baseline −60.6 (*P* > 0.05 since baseline) *GPx* μ 9890 ± 3004 U/L; mean change from baseline 682 U/L (*P* > 0.05 since baseline) *Pro-inflammatory cytokines IL-1β*; *IL-6*; *TNF-α* data not reported (*P* > 0.05 since baseline) *CRP* μ 0.59 ± 0.51 ng/mL; mean change from baseline −0.38 (*P* = 0.05 since baseline) *Haemoglobin* μ 11.0 ± 1.2 g/dL; mean change from baseline −0.1 (*P* > 0.05 since baseline)
Cruciani et al. 2006 [27]	Intervention: Levocarnitine solution (1 g carnitine per mL) Comparator: None Dose: 250 mg, 750 mg, 1250 mg, 1750 mg, 2250 mg, 2750 mg or 3000 mg. Given in two doses/day to meet total reported dosage Duration: 1-week	For all patients (*n* = 27): **Fatigue:** At 1-week post-baseline: *Brief Fatigue Inventory* (scored 0–90; higher scores indicate more severe symptoms) μ 39.7 ± 26.0; mean change from baseline −26.4 (***P* < 0.001 since baseline**) **Mood:** At 1-week post-baseline: *Centre for Epidemiologic Studies Depression Scale* (scored 0–60; higher scores indicate more severe symptoms) μ 19.0 ± 12.0; mean change from baseline −10.2 (***P* < 0.001 since baseline**) **Sleep:** At 1-week post-baseline: *Epworth Sleeplessness Scale* (scored 0–24; higher scores indicate more severe symptoms) μ 9.0 ± 6.0; mean change from baseline −3.9 (***P* = 0.001 since baseline**) **Pathology:** At 1-week post-baseline: *Haemoglobin* μ 12.0 ± 2.0 g/dL; mean change from baseline −0.02 g/dL (***P* = 0.03 since baseline**) **Adverse events:** *n* = 2 mild nausea
Callander et al. 2014 [32]	Intervention: Acetyl-l-carnitine (not further described) Dose: 2 × 1.5 g (3 g per day total) Comparator: IV bortezomib, doxorubicin and oral low-dose dexamethasone (median 3-months) Duration: variable depending on cycles of therapy needed (median 10-months) All patients received IV bortezomib, doxorubicin and oral low-dose dexamethasone	**Fatigue:** At end of treatment: *FACT-Fatigue* (scored 0–65; lower scores indicate more severe symptoms) IG: μ 22.4 ± 11.2; mean change from baseline 7.5 (*P* = 0.114 since baseline) vs. CG: data not reported. Groups not compared
Iwase et al. 2016 [29]	Supplement: Jelly with BCAA, Co-Q10, l-carnitine Dose: Unclear. Either BCAA 1250 mg; Co-Q10 15 mg; l-carnitine 25 mg per day or double that dosage Comparator: Usual care, with recommendations for adequate exercise and relaxation Duration: 3-weeks	**Fatigue:** At 3-weeks post-baseline: *Brief Fatigue Inventory global fatigue score* (scored 0–10; higher scores indicate more severe symptoms) IG: outcome data not reported; mean change from baseline −1.50 ± 2.2 vs. CG: outcome data not reported; mean change from baseline −0.2 ± 2.2 ***P* = 0.025 in change between groups** **Quality of life:** At 3-months post-baseline: *EORTC-QLQ-C30 global health status sub-group* (scoring unclear; lower scores indicate more severe symptoms) IG: outcome data not reported; mean change from baseline −3.4 ± 20.4 vs. CG: outcome data not reported; mean change from baseline 2.7 ± 24.0 *P* = 0.303 change between groups **Mood:** At 3-weeks post-baseline: *Hospital Anxiety and Depression Scale—Anxiety* (scored 0–21; lower scores indicate more severe symptoms) IG: outcome data not reported; mean change from baseline −0.6 ± 1.9 vs. CG: outcome data not reported; mean change from baseline 0.3 ± 1.5 *P* = 0.053 in change between groups *Hospital Anxiety and Depression Scale—Depression* (scored 0–21; lower scores indicate more severe symptoms) IG: outcome data not reported; mean change from baseline 0.6 ± 2.1 vs. CG: outcome data not reported; mean change from baseline −0.1 ± 1.6 *P* = 0.154 change between groups **Adverse events:** Most common severe adverse events were leukopenia and neutropenia. Detailed list of adverse events included in Iwase et al.
Cruciani et al. 2009 [30]	Supplement: l-carnitine syrup (1 g carnitine per 10 mL) Dose: 4-days to progress to 2 × 1 g l-carnitine (2 g carnitine; 10 mL syrup total per day) Comparator: matching placebo Duration: 4-weeks including dose-escalation phase (2 weeks for the CG cross-over participants)	**Fatigue:** At 4-weeks post-baseline: *FACT-Anaemia fatigue sub-scale* (scoring unclear; lower scores indicate more severe symptoms) IG: μ 22.4 ± 10.7; mean change from baseline 6.4 vs. CG: μ 15.1 ± 4.8; mean change from baseline 3.3 ***P* = 0.03 between groups** (adjusted) **Quality of life:** At 4-weeks post-baseline: *FACT-Anaemia physical sub-scale* (scoring unclear; lower scores indicate more severe symptoms) IG: μ 16.5 ± 6.7; mean change from baseline 1.3 vs. CG: μ 14.9 ± 4.0; mean change from baseline 0.9 *P* = 0.12 between groups (adjusted) *FACT-Anaemia social/family sub-scale* (scoring unclear; lower scores indicate more severe symptoms) IG: μ 23.4 ± 6.3; mean change from baseline −0.6 vs. CG: μ 21.3 ± 12.9; mean change from baseline −2.6 *P* = 0.21 between groups (adjusted) *FACT-Anaemia emotional sub-scale* (scoring unclear; lower scores indicate more severe symptoms) IG: μ 14.5 ± 5.8; mean change from baseline 0.3 vs. CG: μ 17.2 ± 4.9 ^Ω^; mean change from baseline 5.6 *P* = 0.35 between groups (adjusted) *FACT-Anaemia function sub-scale* (scoring unclear; lower scores indicate more severe symptoms) IG: μ 11.0 ± 3.2; mean change from baseline −0.4 vs. CG: μ 9.2 ± 3.8; mean change from baseline −1.6 ***P* = 0.002 between groups** (adjusted) *Linear Analogue Scale Assessments* (scoring unclear; lower scores indicate more severe symptoms) IG: μ 37.5 ± 18.3; mean change from baseline 8.6 vs. CG: μ 27.5 ± 19.1; mean change from baseline 6.5 *P* = 0.11 between groups **Physical function:** At 4-weeks post-baseline: *KPS* (scoring 0–100; lower scores indicate more severe symptoms) IG: μ 64.2 ± 9.0; mean change from baseline 6 vs. CG: μ 50.0 ± 15.5; mean change from baseline −7 ***P* = 0.002 between groups** (adjusted) **Adverse events:** *n* = 1 constipation *n* = 1 diarrhoea
Kraft et al. 2012 [22]	Supplement: l-carnitine liquid formulation (not further described) Dose: 4 g/day Comparator: matching placebo Duration: 3-months	**Fatigue:** At 3-months post-baseline: *Brief Fatigue Inventory* (scored 0–90; higher scores indicate more severe symptoms) IG: 28.6% had score >4 vs. CG: 41.7% had score >4 *P* > 0.05 between groups **Anthropometry:** At 6-weeks post-baseline: *Body mass index* via BIA IG: data not reported; mean change from baseline 3.4% ± 1.5% vs. CG: data not reported; mean change from baseline 1.5% ± 1.4% ***P* < 0.018 between groups** **Quality of life:** At 3-months post-baseline: *EORTC-QLQ-C30 global health status sub-group* (scoring unclear; lower scores indicate more severe symptoms) IG: data not reported; mean change from baseline 0.8 vs. CG: data not reported; mean change from baseline −0.3 ***P* < 0.041 between groups** *At 6-weeks post-baseline:* *EORTC-QLQ-C30 cognitive function sub-group* (scoring unclear; lower scores indicate more severe symptoms) IG: data not reported; mean change from baseline 0.3 vs. CG: data not reported; mean change from baseline −0.1 ***P* < 0.034 between groups**
Cruciani et al. 2012 [24]	Supplement: 10 g Levocarnitine inert salt in 100 mL solution Dose: 2 × 1 g l-carnitine per day Comparator: matching placebo Duration: 2-months (1-month for CG cross-over participants)	**Fatigue:** At 4-weeks post-baseline: *Brief Fatigue Inventory* (scored 0–90; higher scores indicate more severe symptoms) IG: data not reported; mean change from baseline −1.0 (**95% CI: −1.3 to −0.6**) vs. CG: data not reported; mean change from baseline −1.1 (**95% CI: −1.4 to 0.8**) *P* = 0.57 between groups *FACT-Fatigue* (scored 0–65; lower scores indicate more severe symptoms) Data not reported. *P* = 0.64 between groups **Mood:** At 4-week post-baseline: *Centre for Epidemiologic Studies Depression Scale* (scored 0–60; higher scores indicate more severe symptoms) Data not reported *P* = 0.93 between groups **Physical function:** At 8-weeks post-baseline *ECOG PS* (scoring 0–5; higher scores indicate more severe symptoms) IG: 18% improved and 18% remained stable vs. CG: 64% improved and 20% remained stable *P* = 0.63 between groups
Hershman et al. 2013 [20]	Supplement: Acetyl-l-carnitine capsules Dose: 6 × 500 mg Acetyl-l-carnitine (3 g per day total) Comparator: matching placebo Duration: 6-months	**Fatigue:** At 6-months post-baseline: *FACT-Fatigue* (scored 0–65; lower scores indicate more severe symptoms) IG: mean change from baseline 1.7 vs. CG: mean change from baseline 2.2 *P* = 0.51 between groups **Functional status:** At 12-weeks post-baseline: *FACT-Taxane Trial Outcome Index* (scoring unclear; lower scores indicate more severe symptoms) IG: μ 91.9; mean change from baseline −7.4 vs. CG: data not reported; mean change from baseline −7.4 At 6-months pose-baseline: IG was 3.5 points lower than CG ***P* = 0.03 between groups** **Adverse events:** IG: grade 3 toxicity (*n* = 3), vomiting (*n* = 1); CG: insomnia (*n* = 1)
Mantovani et al. 2010 [26]	Supplement: l-carnitine (not further described) Dose: 4 g/day Duration: 4-months Comparator: MPA (500 mg/day) or MA (320 mg/day) + eicosapentaenoic acid (EPA) enriched supplement (2.2 g/day) + thalidomide (200 mg/day) + l-carnitine (4 g/day) Other groups (not reported here) were (1) MPA or MA; (2) EPA enriched supplement; (3) thalidomide All patients given: polyphenols 300 mg/day; lipoic acid 300 mg/day; carbocysteine 2.7 g/day; vitamin E 400 mg/day; vitamin A 30,000 IU/day; and vitamin C 500 mg/day	**Fatigue:** At 4-months post-baseline: *MFSI-SF* (scored 0–150; higher scores indicate more severe symptoms) IG-a: μ 26.1 ± 25; mean change from baseline 0.85 ± 19.5 (*P* = 0.801 since baseline) vs. IG-b: μ 20 ± 23.1; mean change from baseline −7.5 ± 12.8 (***P* = 0.047 since baseline**) ***P* = 0.004 between groups** (mean change) **Quality of life:** At 4-months post-baseline: *EORTC-QLQ-C30* (scored 0–100; lower scores indicate more severe symptoms) IG-a: μ 57.1 ± 21; mean change from baseline 1.9 (*P* = 0.832 since baseline) vs. IG-b: μ 65.8 ± 18; mean change from baseline 9.8 (*P* = 0.145 since baseline). Groups not compared *EQ-5D index* (scoring unclear) IG-a: μ 0.4 ± 0.5; mean change from baseline −0.1 (*P* = 0.151 since baseline) vs. IG-b: μ 0.6 ± 0.4; mean change from baseline 0.1 (*P* = 0.092 since baseline). Groups not compared *EQ-5D visual analogue scale* (scored 0–100; lower scores indicate more severe symptoms) IG-a: μ 50.0 ± 26.8; mean change from baseline 4.7 (*P* = 0.593 since baseline) vs. IG-b: μ 49.2 ± 18.0; mean change from baseline −2.5 (*P* = 0.950 since baseline) **Physical function:** At 4-months post-baseline: *ECOG PS* (scoring 0–5; higher scores indicate more severe symptoms) IG-a: μ 1.5 ± 0.9; mean change from baseline −0.4 (***P* = 0.0001 since baseline**) vs. IG-b: μ 1.5 ± 0.8; mean change from baseline −0.5 (***P* < 0.0001 since baseline**). Groups not compared **Anthropometry:** At 4-months post-baseline: *Lean body mass* via BIA IG-a: μ 44.6 ± 8.7 kg; mean change from baseline −0.52 ± 3.14 kg (*P* = 0.952 since baseline) vs. IG-b: μ 44.0 ± 7.2 kg; mean change from baseline 0.44 ± 3.1 kg (*P* = 0.609 since baseline) *P* = 0.144 between groups *Lean body mass* via DEXA IG-a: μ 45.2 ± 16.7 kg; mean change from baseline −0.7 ± 2.2 kg (*P* = 0.980 since baseline) vs. IG-b: μ 44.9 ± 7.7 kg; mean change from baseline 2.1 ± 2.1 kg (***P* = 0.0148 since baseline**) ***P* < 0.001 between groups** *Lean body mass* via CT at L3 IG-a: μ 43.5 ± 29.4 kg; mean change from baseline 1.2 kg (*P* = 0.058 since baseline) vs. IG-b: μ 45.4 ± 23.9 kg; mean change from baseline 2.6 kg (***P* = 0.001 since baseline**). Groups not compared **Muscle strength:** At 4-months post-baseline: *Grip strength* via dynamometer IG-a: μ 25.1 ± 11.9; mean change from baseline −0.8 (*P* = 0.104 since baseline) vs. IG-b: μ 24.2 ± 7.2; mean change from baseline −3 (*P* = 0.399 since baseline). Groups not compared **Appetite:** At 4-months post-baseline: *Visual analogue scale* (scoring unclear; lower scores indicate more severe symptoms) IG-a: μ 5.3 ± 3.1; mean change from baseline 0.2 (*P* = 0.607 since baseline) vs. IG-b: μ 6.1 ± 1.5; mean change from baseline 1.0 (***P* = 0.00037 since baseline**). Groups not compared **Pathology:** At 4-months post-baseline: *IL-6* IG-a: μ 31.6 ± 27.9 pg/mL; mean change from baseline −12.2 pg/mL (*P* = 0.663 since baseline) vs. IG-b: μ 24.7 ± 23.4 pg/mL; mean change from baseline −16.7 pg/mL (***P* = 0.0187 since baseline**). Groups not compared. *TFN-α* IG-a: μ 37.5 ± 40.7 pg/mL; mean change from baseline 5.3 pg/mL (*P* = 0.240 since baseline) vs. IG-b: μ 22.5 ± 21.8 pg/mL; mean change from baseline −14.8 pg/mL (*P* = 0.053 since baseline). Groups not compared *ROS* IG-a: μ 458 ± 138 FORT U; mean change from baseline 9 FORT U (*P* = 0.736 since baseline) vs. IG-b: μ 445 ± 115 FORT U; mean change from baseline −52 FORT U (*P* = 0.262 since baseline). Groups not compared *GPx* IG-a: μ 7107 ± 3398 IU/mL; mean change from baseline 666 IU/mL (*P* = 0.383 since baseline) vs. IG-b: μ 6676 ± 2542 IU/mL; mean change from baseline −758 IU/mL (*P* = 0.816 since baseline). Groups not compared **Adverse events:** IG-a *n* = 1 diarrhoea; IG-b *n* = 1 diarrhoea
Macciò et al. 2012 [25]	Supplement/treatments: MA + l-carnitine + celecoxib + antioxidants (alpha lipoic acid and carbocysteine) Dose: MA 320 mg/day; l-carnitine 4 g/day; alpha lipoic acid 600 mg/day; carbocysteine 2.7 g/day; celecoxib 300 mg/day Comparator: MA (320 mg/day) Duration: 4-months	**Fatigue:** At 4-months post-baseline: *MFSI-SF* (scored 0–150; higher scores indicate more severe symptoms) IG-a: μ 19.9 ± 20.5; mean change from baseline −6.4 (***P* = 0.045 since baseline**) vs. IG-b: μ 23.5 ± 18.2; mean change from baseline 0.9 (*P* = 0.483 since baseline) ***P* = 0.049 between groups** **Quality of life:** At 4-months post-baseline: *EORTC-QLQ-C30* (scored 0–100; lower scores indicate more severe symptoms) IG-a: μ 61.3 ± 20.9; mean change from baseline 7.5 (***P* = 0.029 since baseline**) vs. IG-b: μ 61.1 ± 15.5; mean change from baseline 4.1 (***P* = 0.042 since baseline**) ***P* = 0.042 between groups** **Anthropometry:** At 4-months post-baseline: *Lean body mass* via DEXA IG-a: μ 45.4 ± 10.2 kg; mean change from baseline 2.4 kg (***P* = 0.002 since baseline**) vs. IG-b: μ 45.7 ± 8.2 kg; mean change from baseline 1.3 kg (*P* = 0.584 since baseline) ***P* = 0.032 between groups** **Appetite:** At 4-months post-baseline: *Visual analogue scale* (scoring unclear; lower scores indicate more severe symptoms) IG-a: μ 6.0 ± 1.0; mean change from baseline 1.5 (***P* = 0.019 since baseline**) vs. IG-b: μ 6.3 ± 1.5; mean change from baseline 1.2 (***P* = 0.040 since baseline**) *P* = 0.774 between groups **Muscle strength:** At 4-months post-baseline: *Grip strength* via dynamometer IG-a: μ 27.2 ± 13.9 kg; mean change from baseline 3 (*P* = 0.399 since baseline) vs. IG-b: μ 24.3 ± 8.9; mean change from baseline −1.1 kg (*P* = 0.140 since baseline) *P* = 0.302 between groups **Physical function:** At 4-months post-baseline: *ECOG PS* (scoring 0–5; higher scores indicate more severe symptoms) IG-a: μ 1.1 ± 0.8; mean change from baseline −0.7 (***P* = 0.001 since baseline**) vs. IG-b: μ 1.1 ± 1.2; mean change from baseline −0.5 (***P* = 0.035 since baseline**) *P* = 0.231 between groups **Pathology:** At 4-months post-baseline: *CRP* IG-a: μ 15.3 ± 6.7 mg/L; mean change from baseline −9.2 mg/L (***P* = 0.038 since baseline**) vs. IG-b: μ 21.2 ± 19.7 mg/L; mean change from baseline −7.4 mg/L (*P* = 0.292 since baseline) *P* = 0.056 between groups *SOD* IG-a: μ 96 ± 12; mean change from baseline 11 (*P* = 0.185 since baseline) vs. IG-b: μ 94 ± 15; mean change from baseline 3 (*P* = 0.345 since baseline) *P* = 0.345 between groups *IL-6* IG-a: μ 12.9 ± 10.5 pg/mL; mean change from baseline −9.4 pg/mL (***P* = 0.05 since baseline**) vs. IG-b: μ 28.2 ± 23.8 pg/mL; mean change from baseline 1.0 pg/mL (*P* = 0.622 since baseline) ***P* = 0.003 between groups** *TFN-α* IG-a: μ 21.4 ± 22.6 pg/mL; mean change from baseline −22 pg/mL (***P* = 0.036 since baseline**) vs. IG-b: μ 54.0 ± 25.3 pg/mL; mean change from baseline 13 pg/mL (*P* = 0.829 since baseline) ***P* = 0.04 between groups** *ROS* IG-a: μ 444 ± 71.9 FORT U; mean change from baseline −84 FORT U (***P* = 0.006 since baseline**) vs. IG-b: μ 427 ± 102 FORT U; mean change from baseline −33 FORT U (*P* = 0.092 since baseline) ***P* = 0.037 between groups** *GPx* IG-a: μ 7458 ± 3554 U/L; mean change from baseline 1451 U/L (*P* = 0.233 since baseline) vs. IG-b: μ 7304 ± 5521; mean change from baseline 683 U/L (*P* = 0.320 since baseline) *P* = 0.185 between groups **Adverse events:** IG-a *n* = 2 diarrhoea and *n* = 1 epigastria; IG-b *n* = 1 epigastria
Madeddu et al. 2012 [30]	Supplement/ treatments: l-carnitine + celecoxib Dose: l-carnitine 4 g/day; celecoxib dose not specified Comparator: Dose: MA (320 mg/day); l-carnitine (4 g/day); celecoxib dose not specified Duration: 4-months All patients also had antioxidants polyphenols 300 mg/day; lipoic acid 300 mg/day; carbocysteine 2.7 g/day; vitamin E 400 mg/day; Vitamin A 30,000 IU/day, vitamin C 500 mg/day	**Fatigue:** At 4-months post-baseline: *MFSI-SF* (scored 0–150; higher scores indicate more severe symptoms) IG-a: μ 19.9 ± 16.6; mean change from baseline −7.4 (***P* = 0.036 since baseline**) vs. IG-b: μ 13.5 ± 11.8; mean change from baseline −8.8 (***P* = 0.025 since baseline**) *P* = 0.981 between groups **Quality of life:** At 4-months post-baseline: *EORTC-QLQ-C30* (scored 0–100; lower scores indicate more severe symptoms) IG-a: μ 61.9 ± 16.6; mean change from baseline 1.3 (*P* = 0.333 since baseline) vs. IG-b: μ 70.5 ± 16.2; mean change from baseline 6.6 (*P* = 0.258 since baseline) *P* = 0.514 between groups *ECOG PS* (scoring 0–5; higher scores indicate more severe symptoms) IG-a: μ 1.4 ± 0.7; mean change from baseline −0.4 (***P* = 0.009 since baseline**) vs. IG-b: μ 1.4 ± 0.8; mean change from baseline −0.3 (***P* = 0.030 since baseline**) *P* = 0.796 between groups **Appetite:** At 4-months post-baseline: *Visual analogue scale* (scoring unclear; lower scores indicate more severe symptoms) IG-a: μ 7.6 ± 2.8; mean change from baseline 1.4 (***P* = 0.046 since baseline**) vs. IG-b: μ 7.3 ± 2.3; mean change from baseline 1.4 (***P* = 0.016 since baseline**) *P* = 0.250 between groups **Anthropometry:** At 4-months post-baseline: *Lean body mass* via DEXA IG-a: μ 41.0 ± 9.2 kg; mean change from baseline 2.4 kg (***P* = 0.026 since baseline**) vs. IG-b: μ 43.8 ± 6.4 kg; mean change from baseline 2.5 kg (***P* = 0.036 since baseline**) *P* = 0.333 between groups *Lean body mass* via BIA IG-a: μ 40.9 ± 8.7 kg; mean change from baseline 1.1 kg (*P* = 0.316 since baseline) vs. IG-b: μ 44.6 ± 5.9 kg; mean change from baseline 3.6 kg (*P* = 0.676 since baseline) *P* = 0.407 between groups *Lean body mass* via CT at L3 IG-a: μ 32.4 ± 10.9 kg; mean change from baseline 0.5 kg (***P* = 0.048 since baseline**) vs. IG-b: μ 41.8 ± 8.5 kg; mean change from baseline 1.3 kg (***P* = 0.041 since baseline**) *P* = 0.656 between groups **Muscle strength:** At 4-months post-baseline: *Grip strength* via dynamometer IG-a: μ 29.9 ± 7.8 kg; mean change from baseline 3.8 kg (*P* = 0.140 since baseline) vs. IG-b: μ 29.2 ± 9.1 kg; mean change from baseline 1.7 kg (*P* = 0.380 since baseline) *P* = 0.338 between groups **Physical function:** At 4-months post-baseline: *6-min walk test* IG-a: μ 474 ± 79 m; mean change from baseline 45 m (***P* = 0.015 since baseline**) vs. IG-b: μ 464 ± 97 m; mean change from baseline 53 m (***P* = 0.038 since baseline**) *P* = 0.626 between groups **Pathology:** At 4-months post-baseline: *CRP* IG-a: μ 21.2 ± 19.7 mg/L; mean change from baseline −7.8 mg/L (*P* = 0.291 since baseline) vs. IG-b: μ 10.3 ± 11.6 mg/L; mean change from baseline −11.5 mg/L (*P* = 0.239 since baseline) *P* = 0.840 between groups *IL-6* IG-a: μ 20.6 ± 17.8 pg/mL; mean change from baseline −4.1 pg/mL (*P* = 0.543 since baseline) vs. IG-b: μ 19.4 ± 29.2 pg/mL; mean change from baseline −3 pg/mL (*P* = 0.781 since baseline) *P* = 0.877 between groups *TFN-α* IG-a: μ 26.4 ± 5.2 pg/mL; mean change from baseline −0.6 pg/mL (*P* = 0.829 since baseline) vs. IG-b: μ 26.5 ± 6.7 pg/mL; mean change from baseline −1.1 pg/mL (*P* = 0.475 since baseline) *P* = 0.548 between groups **Adverse events** IG-a: diarrhoea (*n* = 1), epigastria (*n* = 1); IG-b: diarrhoea (*n* = 1), epigastria (*n* = 1)

μm, micrometre; BCAA, branched chain amino acid; BIA, bioelectrical impedance analysis; CG, control group; Co-Q10, coenzyme Q10; COI, conflict of interest; CRP, c-reactive protein; CT, computed tomography; DEXA, dual-energy X-ray absorptiometry; ECOG PS, decilitre; Eastern Cooperative Oncology Group performance status; dL, decilitre; FACT, Functional Assessment of Cancer Therapy; g, gram; GPx, Glutathione peroxidase; IG, intervention group; IL, interleukin; kcal, kilocalorie; IU, international unit; IV, intravenous; kg, kilogram; KPS, Karnofsky Performance Status; L, litre; L3, third lumbar vertebrae; m, meter; megestrol acetate; MPA, medroxyprogesterone acetate; MFSI-SF, Multidimensional Fatigue Symptoms Inventory-Short Form; mg, milligrams; MPA, medroxyprogesterone acetate; N/A, not applicable; pg, picogram; QoL, quality of life; ROS, Reactive oxygen species; SOD, superoxide dismutase; TNF, tumour necrosis factor. ^Ω^ Data reported in Cruciani et al. 2009 [23] was a mean of 117.2 ± 4.9; however, due to the scoring of the assessment tool sub-scale and other data points reported in using this tool the review authors believe this to be an error and that the mean was 17.2.
